# Supplementary material for: Impacts of Urban Green on Cardiovascular and Cerebrovascular Diseases—A Systematic Review and Meta-Analysis
Source: Int J Environ Res Public Health. 2023 May 26;20(11):5966. doi: 10.3390/ijerph20115966 (PMC10253108; doi:10.3390/ijerph20115966)
Supplement: Supplementary file 1 [file ijerph-20-05966-s001.zip › ijerph-2425378-supplementary.pdf]

SUPPLEMENTAL MATERIAL

1. Supplementary data extraction

Supplementary data extractions and quality score assessment

Authors	Year	Title	Journal	Country	Co-variates
Richardson et al. [14]	2010	Gender differences in relationships between urban green space and health in the United Kingdom	Soc. Sci. Med.	UK	age-group, income deprivation, country and air pollution
Richardson et al. [15]	2010	The association between green space and cause-specific mortality in urban New Zealand: An ecological analysis of green space utility	BMC Public Health	New Zeland	Sex, Age, Area Deprivation, Smoking rate, Air Pollution
Richardson et al. [16]	2012	Green cities and health: A question of scale?	J. Epidemiol. Community Health	USA	socioeconomic deprivation, ethnicity, PM10, and car dependency
Pereira et al. [17]	2012	The association between neighborhood greenness and cardiovascular disease: An observational study	BMC Public Health	Australia	age, sex, education, income, possession of a healthcare card, diabetes, high cholesterol, high blood pressure, BMI, fruit consumption, vegetable consumption, high-risk alcohol consumption, smoking, air quality (total length of main roads)

Villeneuve et al. [18]	2012	A cohort study relating urban green space with mortality in Ontario, Canada	Environ. Res.	Canada	income, age, sex and marital status, income, unemployment, immigration, exposure to ambient vehicular pollution, indirect adjustment for cigarette smoking
Richardson et al. [19]	2013	Role of physical activity in the relationship between urban green space and health	Public Health	New Zeland	Age, Sex, Ethnicity, NZiDep, Smoking
Tamosiunas et al. [20]	2014	Accessibility and use of urban green spaces, and cardiovascular health: Findings from a Kaunas cohort study	Environ. Health Global Access Sci. Sour.	Lithuania	age, sex, education, smoking, arterial hypertension, physical activity, total cholesterol level, fasting glucose level, body mass index, diabetes mellitus, cognitive function, symptoms of depression, self-rated health, and quality of life.
Bixby et al. [21]	2015	Associations between green space and health in english cities: An ecological, cross-sectional study	PLoS ONE	UK	income deprivation and PM10 concentration
Massa et al. [22]	2016	Environmental factors and cardiovascular diseases: The association of income inequality and green spaces in elderly residents of São Paulo, Brazil	BMJ Open	Brazil	age, gender, ethnicity, education, income, marital status, alcohol ingestion, smoking, BMI, diabetes, hypertension, area-level average income
Xu et al. [23]	2017	An ecological study of the association between area-level green space and adult mortality in Hong Kong	Clim.	Hong Kong	age, gender, education, median household income, occupation

Wang et al. [24]	2017	Neighbouring green space and mortality in community-dwelling elderly Hong Kong Chinese: A cohort study	BMJ Open	China	age, sex, marital status, years lived in Hong Kong, education level, socioeconomic ladder, smoking, alcohol intake, diet quality, self-rated health and housing type, physical activity (PASE) and cognitive function (MMSE).
Crouse et al. [25]	2017	Urban greenness and mortality in Canada's largest cities: a national cohort study	Lancet Planet. Health	Canada	age, sex, census metropolitan area, visible minority status, Aboriginal identity, marital status, highest level of education, income quintile, labour force status, employment, education, income, population density, PM2.5, O3, NO2.
da Silveira et al. [26]	2017	Green spaces and mortality due to cardiovascular diseases in the city of Rio de Janeiro	Rev. Saude Publica	Brazil	age, gender, Social Development Index, light and heavy road densities, indicator of coastal sectors
Vienneau et al. [27]	2017	More than clean air and tranquillity: Residential green is independently associated with decreasing mortality	Environ. Int.	Switzerland	age, sex, civil status, job position, educational attainment, SEP, region, area type, altitude, PM10, total transportation noise

Servadio et al. [28]	2019	Demographic Inequities in Health Outcomes and Air Pollution Exposure in the Atlanta Area and its Relationship to Urban Infrastructure	Urban Health	USA	air pollution, the indicator variable of having a predominantly African-American population, the percentage of elderly residents, park access, tree canopy cover, road intersection connectivity, and value of construction projects
Orioli et al. [29]	2019	Exposure to residential greenness as a predictor of cause-specific mortality and stroke incidence in the rome longitudinal study	Environ. Health Perspect.	Italy	age, sex, educational level, marital status, occupational status, place of birth, area-level socioeconomic position
Seo et al. [30]	2019	Association between urban green space and the risk of cardiovascular disease: A longitudinal study in seven Korean metropolitan areas	Environ. Int.	South Korea	age, sex, income, disability, and Charlson comorbidity index
Wang et al. [31]	2019	Effects of greenspace morphology on mortality at the neighbourhood level: a cross-sectional ecological study	Lancet Planet. Health	USA	age, gender, ethnic origin, education, and income
Jennings et al. [32]	2019	Structural Characteristics of Tree Cover and the Association with Cardiovascular and Respiratory Health in Tampa, FL	Urban Health	USA	Income, percent owner, population density, ethnicity
Astell-Burt et al. [10]	2019	Urban green space, tree canopy and prevention of cardiometabolic diseases: A multilevel longitudinal study of 46 786 Australians	Int. J. Epidemiol.	Australia	age, sex, annual household income, highest educational qualification, economic status, marital status
Kim et al. [33]	2019	Interactions between ambient air particles and greenness on cause-specific mortality in seven Korean metropolitan cities, 2008-2016	Int. J. Environ. Res. Public Health	South Korea	Socioeconomic status, smoking rates, and healthcare infrastructure status, PM10

Paul et al. [34]	2020	Urban green space and the risks of dementia and stroke	Environ. Res.	Canada	Age, sex, income, proportion of recent immigrants, unemployment rate, proportion with less than high school education, coronary heart disease, diabetes, hypertension, congestive heart failure, arrhythmia, and traumatic brain injury, NO2, PM2.5, and O3
Hartig et al. [35]	2020	Associations between greenspace and mortality vary across contexts of community change: A longitudinal ecological study	J. Epidemiol. Community Health	Sweden	gender, age (calculated: gender by age), aggregate values for mean individual disposable income, education, place of birth (Sweden or foreign)
Astell-Burt et al. [36]	2020	Green space and cardiovascular health in people with type 2 diabetes	Health Place	Australia	sex, age group, couple status, highest educational qualification, annual household income before tax, economic status and country or region of birth
Chen et al. [37]	2020	Residential greenness and cardiovascular disease incidence, readmission, and mortality	Environ. Health Perspect.	Canada	age, sex, region, area-level unemployment, percent less than high school education, percent recent immigrants, and household income and population density, NO2 and PM2.5, comorbidities

Yang et al. [38]	2020	Association Between Residential Greenness, Cardiometabolic Disorders, and Cardiovascular Disease Among Adults in China	JAMA Netw Open	China	age, sex, ethnicity, household income, educational level, district-level gross domestic product, physical activity level, and air pollution level
Lee et al. [39]	2020	Association between surrounding greenness and mortality: An ecological study in taiwan	Int. J. Environ. Res. Public Health	Taiwan	total population, age, sex ratio, taxable income, precipitation, time trend, and temperature
Bauwelinck et al. [40]	2021	Residing in urban areas with higher green space is associated with lower mortality risk: A census-based cohort study with ten years of follow-up	Environ. Int.	Belgium	age, sex, marital status, country of birth, education, employment status, area mean income, are unemployment rate and area percentage of population with low (i.e. no/primary) education, air pollution (PM2.5, PM10, NO2 and BC, one at a time)
Padmaka Silva et al. [41]	2021	Associations between residential greenness and self-reported heart disease in Sri Lankan men: A cross-sectional study	PLoS ONE	Sri Lanka	age, marital status, income, education, alcohol consumption, smoking and road length
Liu et al. [42]	2021	Association of neighborhood greenness exposure with cardiovascular diseases and biomarkers	Int. J. Hyg. Environ. Health	China	age, gender, education, and income
Cheruvalath et al. [43]	2022	Associations Between Residential Greenspace, Socioeconomic Status, and Stroke: A Matched Case-Control Study.	Journal of patient-centered research and reviews	USA	Controls matched to cases based on admission year, age, gender, ethnicity, hypertension, diabetes, current or past smoking history, body mass index, and cholesterol

Wang et al. [44]	2022	Analysis of Spatial Distribution of CVD and Multiple Environmental Factors in Urban Residents	Comput. Intell. Neurosci.	China	None
Li et al. [45]	2022	Community built environment and the associated ischemic heart disease risk: Evidence from multi-source data in Wuhan, China.	J. Transp. Health	China	Population density, people over 60yo percentage, Community year (average building age), Housing price, Floor-area ration (FAR), Open space (OP)
Li et al. [46]	2022	Residential greenness, air pollution, and incident ischemic heart disease: A prospective cohort study in China	Sci. Total Environ.	China	age, sex, marital status, education level, income, BMI, smoking, alcohol consumption, and tea consumption
Ponjoan et al. [47]	2022	Impact of residential greenness on myocardial infarction in the population with diabetes: A sex-dependent association?	Environ. Res.	Spain	age, sex, body mass index, hypertension, dyslipidemia,band tobacco consumption, deprivation MEDEA index, air pollution, daytime traffic noise levels and walkability
Ho et al. [48]	2022	Community planning for a “healthy built environment” via a human-environment nexus? A multifactorial assessment of environmental characteristics and age-specific stroke mortality in Hong Kong	Chemosphere	Hong-Kong	Regional air pollution, Traffic-related air pollution, High temperature days, Low temperature days, Daily PM10 , Daily NOx, Daily O3, Relative humidity, Percent of open space, Percent of low education, Percent of non-Cantonese, Unemployed, Unmarried, Sex, Age

2. *Sensitivity analyses*

2.1. Sensitivity analyses - CVD Mortality

Study or Subgroup	log[Hazard Ratio]	SE	Weight	Hazard Ratio IV, Random, 95% CI
Bauwelinck 2021	−0.0101	0.0104	0.0%	0.99 [0.97, 1.01]
Chen 2020	−0.0943	0.0056	20.4%	0.91 [0.90, 0.92]
Crouse 2017	−0.0932	0.0096	19.6%	0.91 [0.89, 0.93]
Orioli 2019	−0.0161	0.0047	20.5%	0.98 [0.98, 0.99]
Vienneau 2017	−0.0726	0.0055	20.4%	0.93 [0.92, 0.94]
Villeneuve 2012	−0.0619	0.011	19.2%	0.94 [0.92, 0.96]
Total (95% CI)			100.0%	0.93 [0.90, 0.97]

Heterogeneity: $Tau^2 = 0.00$; $Chi^2 = 141.92$, df = 4 ($P < 0.00001$); $I^2 = 97\%$
Test for overall effect: Z = 3.89 ($P < 0.0001$)

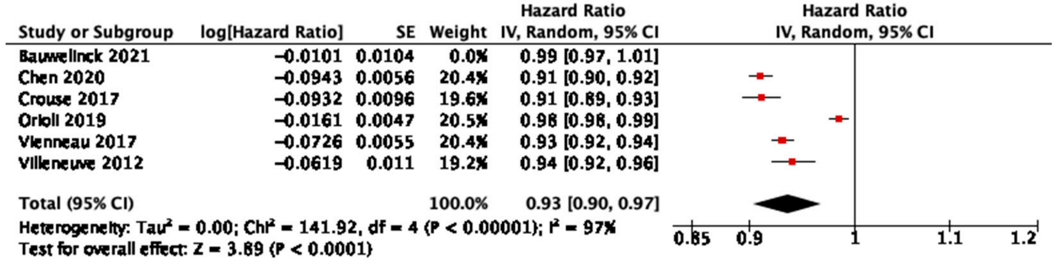

Study or Subgroup	log[Hazard Ratio]	SE	Weight	Hazard Ratio IV, Random, 95% CI
Bauwelinck 2021	−0.0101	0.0104	19.5%	0.99 [0.97, 1.01]
Chen 2020	−0.0943	0.0056	0.0%	0.91 [0.90, 0.92]
Crouse 2017	−0.0932	0.0096	19.7%	0.91 [0.89, 0.93]
Orioli 2019	−0.0161	0.0047	20.8%	0.98 [0.98, 0.99]
Vienneau 2017	−0.0726	0.0055	20.6%	0.93 [0.92, 0.94]
Villeneuve 2012	−0.0619	0.011	19.3%	0.94 [0.92, 0.96]
Total (95% CI)			100.0%	0.95 [0.92, 0.98]

Heterogeneity: $Tau^2 = 0.00$; $Chi^2 = 101.79$, df = 4 ($P < 0.00001$); $I^2 = 96\%$
Test for overall effect: Z = 3.05 ($P = 0.002$)

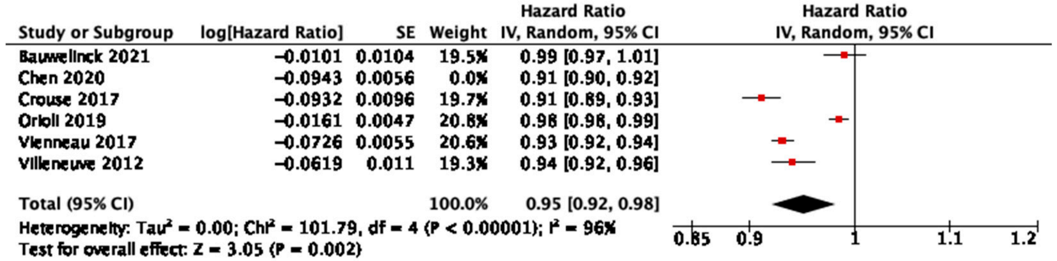

Study or Subgroup	log[Hazard Ratio]	SE	Weight	Hazard Ratio IV, Random, 95% CI
Bauwelinck 2021	−0.0101	0.0104	19.4%	0.99 [0.97, 1.01]
Chen 2020	−0.0943	0.0056	20.4%	0.91 [0.90, 0.92]
Crouse 2017	−0.0932	0.0096	0.0%	0.91 [0.89, 0.93]
Orioli 2019	−0.0161	0.0047	20.5%	0.98 [0.98, 0.99]
Vienneau 2017	−0.0726	0.0055	20.4%	0.93 [0.92, 0.94]
Villeneuve 2012	−0.0619	0.011	19.3%	0.94 [0.92, 0.96]
Total (95% CI)			100.0%	0.95 [0.92, 0.98]

Heterogeneity: $Tau^2 = 0.00$; $Chi^2 = 146.39$, df = 4 ($P < 0.00001$); $I^2 = 97\%$
Test for overall effect: Z = 2.88 ($P = 0.004$)

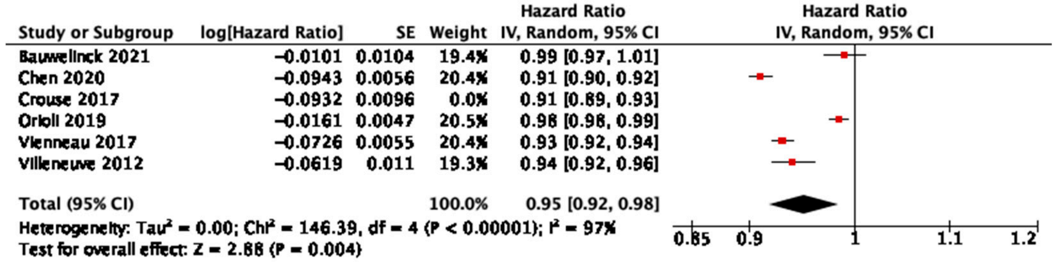

Study or Subgroup	log[Hazard Ratio]	SE	Weight	Hazard Ratio IV, Random, 95% CI
Bauwelinck 2021	−0.0101	0.0104	19.2%	0.99 [0.97, 1.01]
Chen 2020	−0.0943	0.0056	21.1%	0.91 [0.90, 0.92]
Crouse 2017	−0.0932	0.0096	19.6%	0.91 [0.89, 0.93]
Orioli 2019	−0.0161	0.0047	0.0%	0.98 [0.98, 0.99]
Vienneau 2017	−0.0726	0.0055	21.1%	0.93 [0.92, 0.94]
Villeneuve 2012	−0.0619	0.011	19.0%	0.94 [0.92, 0.96]
Total (95% CI)			100.0%	0.94 [0.91, 0.96]

Heterogeneity: $Tau^2 = 0.00$; $Chi^2 = 56.02$, df = 4 ($P < 0.00001$); $I^2 = 93\%$
Test for overall effect: Z = 5.17 ($P < 0.00001$)

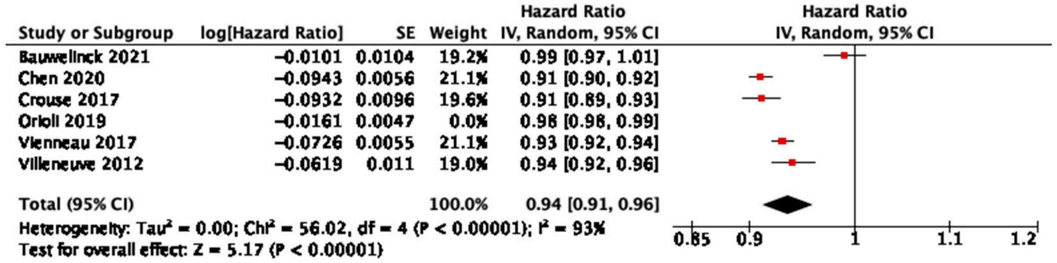

Study or Subgroup	log[Hazard Ratio]	SE	Weight	Hazard Ratio IV, Random, 95% CI
Bauwelinck 2021	−0.0101	0.0104	19.7%	0.99 [0.97, 1.01]
Chen 2020	−0.0943	0.0056	20.4%	0.91 [0.90, 0.92]
Crouse 2017	−0.0932	0.0096	19.8%	0.91 [0.89, 0.93]
Orioli 2019	−0.0161	0.0047	20.5%	0.98 [0.98, 0.99]
Vienneau 2017	−0.0726	0.0055	0.0%	0.93 [0.92, 0.94]
Villeneuve 2012	−0.0619	0.011	19.6%	0.94 [0.92, 0.96]
Total (95% CI)			100.0%	0.95 [0.91, 0.98]

Heterogeneity: $Tau^2 = 0.00$; $Chi^2 = 150.69$, df = 4 ($P < 0.00001$); $I^2 = 97\%$
Test for overall effect: Z = 2.72 (P = 0.007)

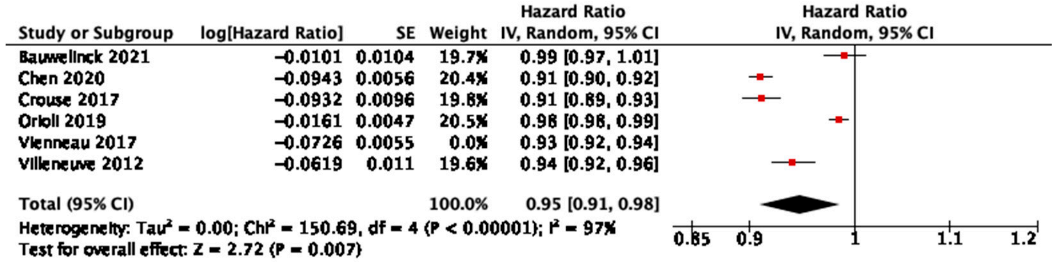

Study or Subgroup	log[Hazard Ratio]	SE	Weight	Hazard Ratio IV, Random, 95% CI
Bauwelinck 2021	−0.0101	0.0104	19.4%	0.99 [0.97, 1.01]
Chen 2020	−0.0943	0.0056	20.3%	0.91 [0.90, 0.92]
Crouse 2017	−0.0932	0.0096	19.6%	0.91 [0.89, 0.93]
Orioli 2019	−0.0161	0.0047	20.4%	0.98 [0.98, 0.99]
Vienneau 2017	−0.0726	0.0055	20.3%	0.93 [0.92, 0.94]
Villeneuve 2012	−0.0619	0.011	0.0%	0.94 [0.92, 0.96]
Total (95% CI)			100.0%	0.94 [0.91, 0.98]

Heterogeneity: $Tau^2 = 0.00$; $Chi^2 = 162.42$, df = 4 ($P < 0.00001$); $I^2 = 98\%$
Test for overall effect: Z = 3.12 (P = 0.002)

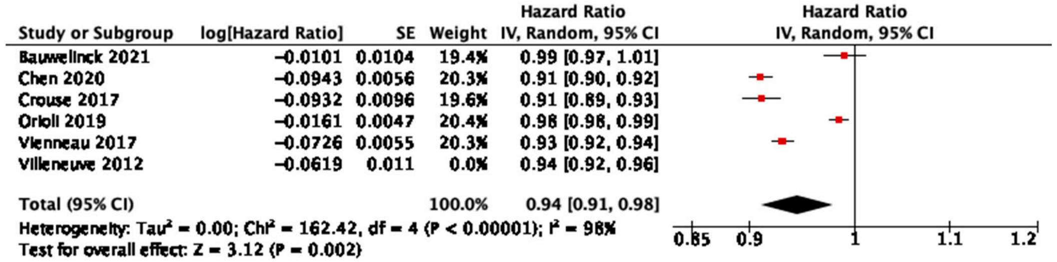

2.2. Sensitivity analyses – IHD Mortality

Study or Subgroup	log[Hazard Ratio]	SE	Weight	Hazard Ratio IV, Random, 95% CI	Year
Villeneuve 2012	−0.0357	0.0079	0.0%	0.96 [0.95, 0.98]	2012
Crouse 2017	−0.1009	0.0126	25.3%	0.90 [0.88, 0.93]	2017
Vienneau 2017	−0.0513	0.0109	25.8%	0.95 [0.93, 0.97]	2017
Orioli 2019	−0.0151	0.0089	26.4%	0.99 [0.97, 1.00]	2019
Bauwelinck 2021	0.0198	0.0204	22.5%	1.02 [0.98, 1.06]	2021
Total (95% CI)			100.0%	0.96 [0.92, 1.01]	

Heterogeneity: $Tau^2 = 0.00$; $Chi^2 = 40.84$, df = 3 ($P < 0.00001$); $I^2 = 93\%$
Test for overall effect: Z = 1.70 (P = 0.09)

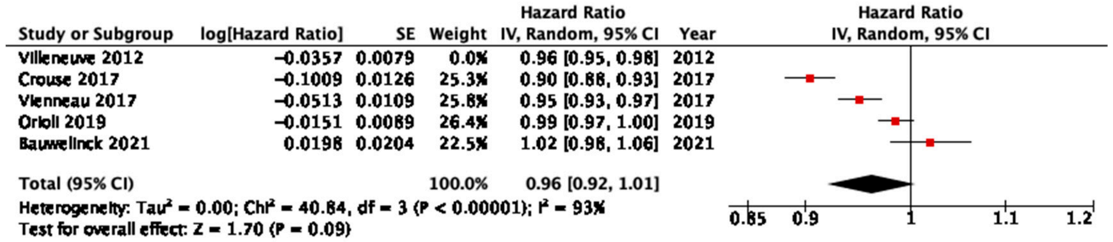

Study or Subgroup	log[Hazard Ratio]	SE	Weight	Hazard Ratio IV, Random, 95% CI	Year
Villeneuve 2012	−0.0357	0.0079	29.4%	0.96 [0.95, 0.98]	2012
Crouse 2017	−0.1009	0.0126	0.0%	0.90 [0.88, 0.93]	2017
Vienneau 2017	−0.0513	0.0109	26.0%	0.95 [0.93, 0.97]	2017
Orioli 2019	−0.0151	0.0089	28.3%	0.99 [0.97, 1.00]	2019
Bauwelinck 2021	0.0198	0.0204	16.2%	1.02 [0.98, 1.06]	2021
Total (95% CI)			100.0%	0.98 [0.95, 1.00]	

Heterogeneity: $Tau^2 = 0.00$; $Chi^2 = 13.07$, df = 3 ($P = 0.004$); $I^2 = 77\%$
Test for overall effect: Z = 2.20 (P = 0.03)

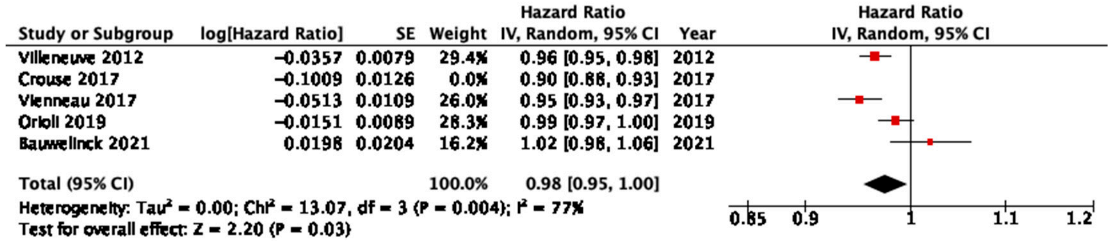

Study or Subgroup	log[Hazard Ratio]	SE	Weight	Hazard Ratio IV, Random, 95% CI	Year
Villeneuve 2012	−0.0357	0.0079	26.7%	0.96 [0.95, 0.98]	2012
Crouse 2017	−0.1009	0.0126	25.1%	0.90 [0.88, 0.93]	2017
Vienneau 2017	−0.0513	0.0109	0.0%	0.95 [0.93, 0.97]	2017
Orioli 2019	−0.0151	0.0089	26.4%	0.99 [0.97, 1.00]	2019
Bauwelinck 2021	0.0198	0.0204	21.7%	1.02 [0.98, 1.06]	2021
Total (95% CI)			100.0%	0.97 [0.93, 1.00]	

Heterogeneity: Tau² = 0.00; Chi² = 39.53, df = 3 (P < 0.00001); I² = 92%
Test for overall effect: Z = 1.71 (P = 0.09)

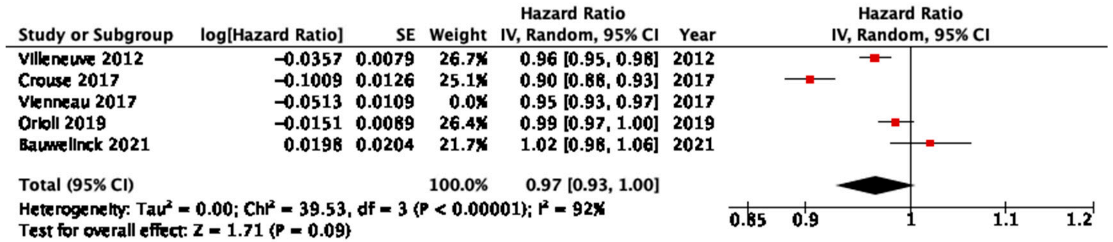

Study or Subgroup	log[Hazard Ratio]	SE	Weight	Hazard Ratio IV, Random, 95% CI	Year
Villeneuve 2012	−0.0357	0.0079	27.1%	0.96 [0.95, 0.98]	2012
Crouse 2017	−0.1009	0.0126	25.3%	0.90 [0.88, 0.93]	2017
Vienneau 2017	−0.0513	0.0109	26.0%	0.95 [0.93, 0.97]	2017
Orioli 2019	−0.0151	0.0089	0.0%	0.99 [0.97, 1.00]	2019
Bauwelinck 2021	0.0198	0.0204	21.5%	1.02 [0.98, 1.06]	2021
Total (95% CI)			100.0%	0.96 [0.92, 0.99]	

Heterogeneity: Tau² = 0.00; Chi² = 31.19, df = 3 (P < 0.00001); I² = 90%
Test for overall effect: Z = 2.32 (P = 0.02)

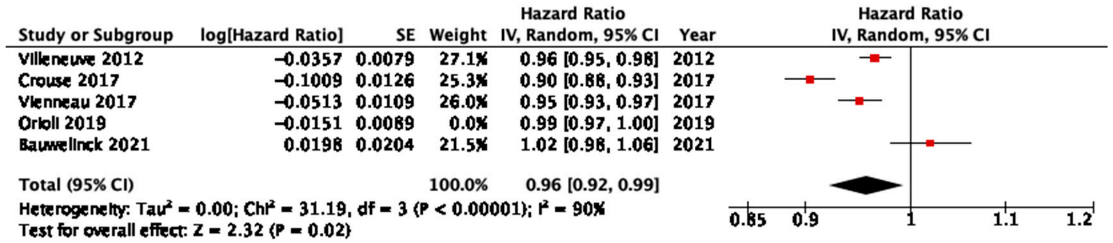

Study or Subgroup	log[Hazard Ratio]	SE	Weight	Hazard Ratio IV, Random, 95% CI	Year
Villeneuve 2012	−0.0357	0.0079	26.0%	0.96 [0.95, 0.98]	2012
Crouse 2017	−0.1009	0.0126	23.7%	0.90 [0.88, 0.93]	2017
Vienneau 2017	−0.0513	0.0109	24.6%	0.95 [0.93, 0.97]	2017
Orioli 2019	−0.0151	0.0089	25.6%	0.99 [0.97, 1.00]	2019
Bauwelinck 2021	0.0198	0.0204	0.0%	1.02 [0.98, 1.06]	2021
Total (95% CI)			100.0%	0.95 [0.92, 0.98]	

Heterogeneity: Tau² = 0.00; Chi² = 32.35, df = 3 (P < 0.00001); I² = 91%
Test for overall effect: Z = 3.08 (P = 0.002)

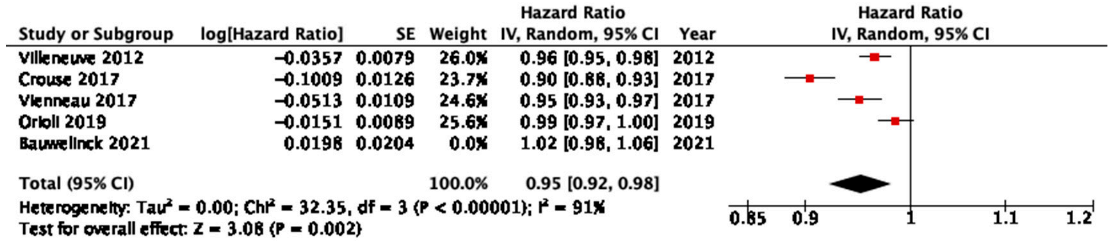

2.3. Sensitivity analyses – CBVD Mortality

Study or Subgroup	log[Hazard Ratio]	SE	Weight	Hazard Ratio IV, Random, 95% CI	Year
Villeneuve 2012	−0.0453	0.0117	0.0%	0.96 [0.93, 0.98]	2012
Vienneau 2017	−0.0726	0.0224	19.4%	0.93 [0.89, 0.97]	2017
Crouse 2017	−0.0598	0.0221	19.8%	0.94 [0.90, 0.98]	2017
Orioli 2019	−0.0356	0.0112	39.6%	0.97 [0.94, 0.99]	2019
Bauwelinck 2021	−0.0101	0.021	21.2%	0.99 [0.95, 1.03]	2021
Total (95% CI)			100.0%	0.96 [0.94, 0.98]	

Heterogeneity: Tau² = 0.00; Chi² = 5.10, df = 3 (P = 0.16); I² = 41%
Test for overall effect: Z = 3.52 (P = 0.0004)

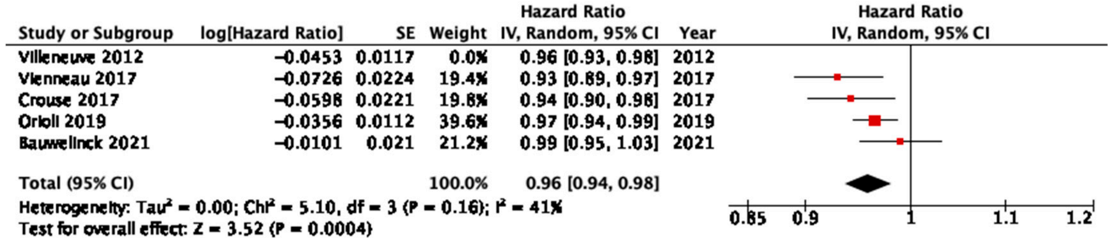

Study or Subgroup	log[Hazard Ratio]	SE	Weight	Hazard Ratio IV, Random, 95% CI	Year
Villeneuve 2012	−0.0453	0.0117	36.9%	0.96 [0.93, 0.98]	2012
Vienneau 2017	−0.0726	0.0224	0.0%	0.93 [0.89, 0.97]	2017
Crouse 2017	−0.0598	0.0221	11.0%	0.94 [0.90, 0.98]	2017
Orioli 2019	−0.0356	0.0112	40.0%	0.97 [0.94, 0.99]	2019
Bauwelinck 2021	−0.0101	0.021	12.1%	0.99 [0.95, 1.03]	2021
Total (95% CI)			100.0%	0.96 [0.95, 0.98]	

Heterogeneity: $Tau^2 = 0.00$; $Chi^2 = 3.16$, df = 3 ($P = 0.37$); $I^2 = 5\%$
Test for overall effect: $Z = 5.22$ ($P < 0.00001$)

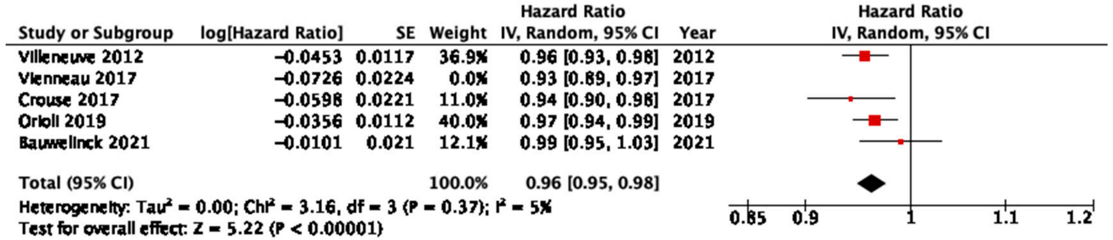

Study or Subgroup	log[Hazard Ratio]	SE	Weight	Hazard Ratio IV, Random, 95% CI	Year
Villeneuve 2012	−0.0453	0.0117	34.4%	0.96 [0.93, 0.98]	2012
Vienneau 2017	−0.0726	0.0224	14.0%	0.93 [0.89, 0.97]	2017
Crouse 2017	−0.0598	0.0221	0.0%	0.94 [0.90, 0.98]	2017
Orioli 2019	−0.0356	0.0112	36.0%	0.97 [0.94, 0.99]	2019
Bauwelinck 2021	−0.0101	0.021	15.6%	0.99 [0.95, 1.03]	2021
Total (95% CI)			100.0%	0.96 [0.94, 0.98]	

Heterogeneity: $Tau^2 = 0.00$; $Chi^2 = 4.50$, df = 3 ($P = 0.21$); $I^2 = 33\%$
Test for overall effect: $Z = 4.32$ ($P < 0.0001$)

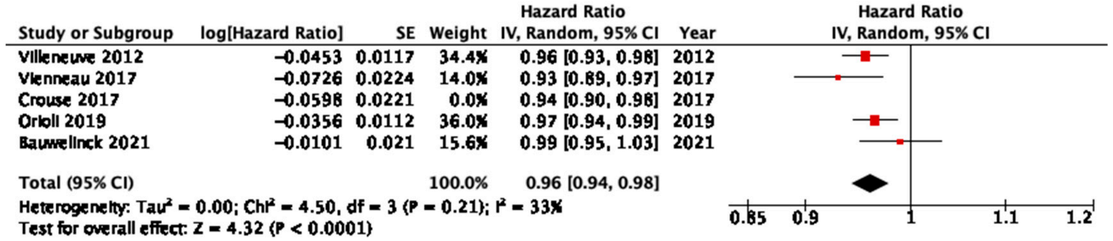

Study or Subgroup	log[Hazard Ratio]	SE	Weight	Hazard Ratio IV, Random, 95% CI	Year
Villeneuve 2012	−0.0453	0.0117	40.2%	0.96 [0.93, 0.98]	2012
Vienneau 2017	−0.0726	0.0224	19.2%	0.93 [0.89, 0.97]	2017
Crouse 2017	−0.0598	0.0221	19.6%	0.94 [0.90, 0.98]	2017
Orioli 2019	−0.0356	0.0112	0.0%	0.97 [0.94, 0.99]	2019
Bauwelinck 2021	−0.0101	0.021	21.0%	0.99 [0.95, 1.03]	2021
Total (95% CI)			100.0%	0.96 [0.93, 0.98]	

Heterogeneity: $Tau^2 = 0.00$; $Chi^2 = 4.72$, df = 3 ($P = 0.19$); $I^2 = 36\%$
Test for overall effect: $Z = 3.97$ ($P < 0.0001$)

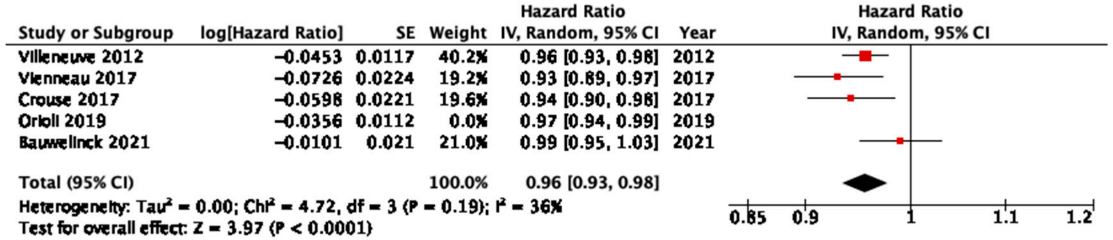

Study or Subgroup	log[Hazard Ratio]	SE	Weight	Hazard Ratio IV, Random, 95% CI	Year
Villeneuve 2012	−0.0453	0.0117	37.8%	0.96 [0.93, 0.98]	2012
Vienneau 2017	−0.0726	0.0224	10.3%	0.93 [0.89, 0.97]	2017
Crouse 2017	−0.0598	0.0221	10.6%	0.94 [0.90, 0.98]	2017
Orioli 2019	−0.0356	0.0112	41.3%	0.97 [0.94, 0.99]	2019
Bauwelinck 2021	−0.0101	0.021	0.0%	0.99 [0.95, 1.03]	2021
Total (95% CI)			100.0%	0.96 [0.94, 0.97]	

Heterogeneity: $Tau^2 = 0.00$; $Chi^2 = 2.66$, df = 3 ($P = 0.45$); $I^2 = 0\%$
Test for overall effect: $Z = 6.34$ ($P < 0.00001$)

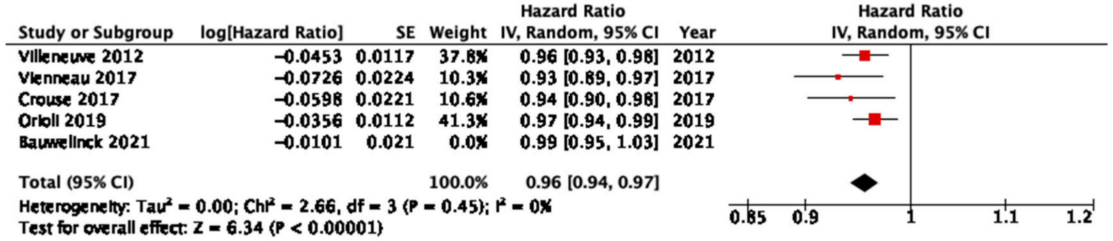

3. Funnel plots

3.1. Funnel plot – CVD mortality

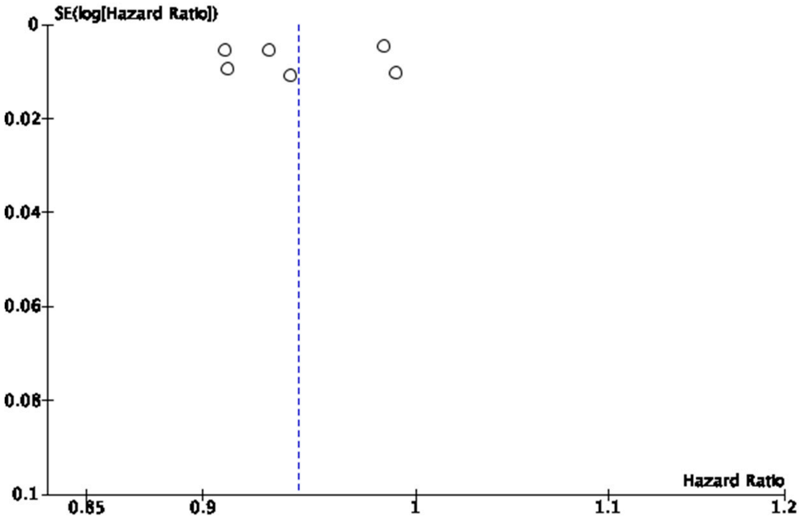

3.2. Funnel plot – IHD mortality

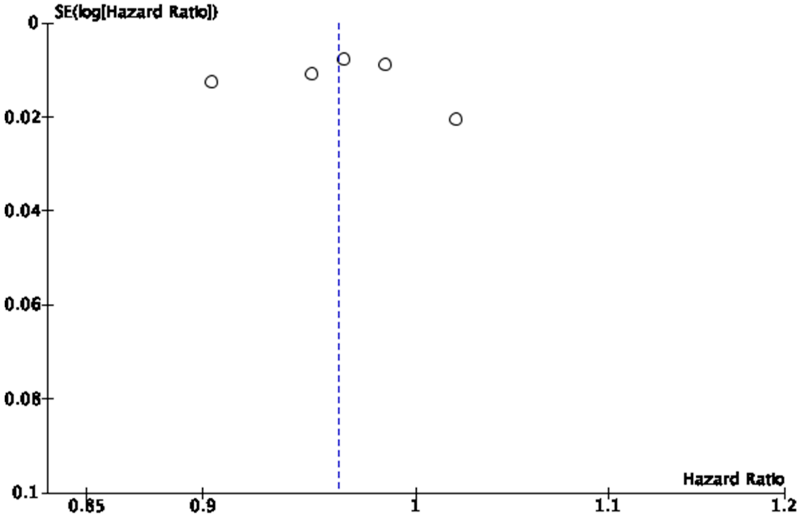

3.3. Funnel plot – CBVD mortality

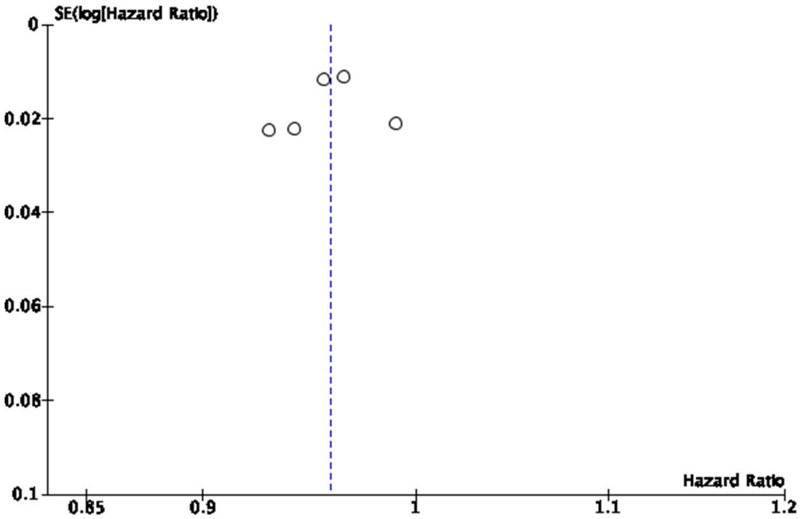
SE(log[Hazard Ratio])
0
0.02
0.04
0.06
0.08
0.1
0.85
0.9
1
1.1
1.2
Hazard Ratio
